# The Effect of Small Size Uterine Fibroids on Pregnancy Outcomes in High-risk Pregnancies

**DOI:** 10.1055/s-0040-1713913

**Published:** 2020-09-29

**Authors:** Murat Cagan, Atakan Tanacan, Hanife Guler Donmez, Erdem Fadiloglu, Canan Unal, Mehmet Sinan Beksac

**Affiliations:** 1 Division of Perinatology, Department of Obstetrics and Gynecology, Ankara, Turkey; 2 Department of Biology, Hacettepe University, Ankara, Turkey

**Keywords:** uterine fibroid, myoma, pregnancy, CS myomectomy

## Abstract

**Objective** To evaluate the obstetric outcomes of singleton high-risk pregnancies with a small size uterine fibroid.

**Methods** This retrospective cohort study was conducted among 172 high-risk pregnant women who were followed-up by a single surgeon between 2016 and 2019. Pregnant women with preconceptionally diagnosed small size (< 5 cm) single uterine fibroids ( *n* = 25) were compared with pregnant women without uterine fibroids ( *n* = 147) in terms of obstetric outcomes.

**Results** There was no statistically significant difference between the groups in terms of adverse pregnancy outcomes. The size of the fibroids was increased in 60% of the cases, and the growth percentage of the fibroids was 25% during pregnancy. Intrapartum and short-term complication was not observed in women who underwent cesarean myomectomy.

**Conclusion** Small size uterine fibroids seem to have no adverse effect on pregnancy outcomes even in high-risk pregnancies, and cesarean myomectomy may be safely performed in properly selected cases.

## Introduction

Uterine fibroids (also known as myomas) are benign monoclonal smooth muscle neoplasms and are the most common pelvic tumors in women of reproductive age. The incidence of fibroids in pregnancy is from 3.3 to 10.7%. 1 2 Although most pregnancies in women with fibroids are uneventful, adverse pregnancy outcomes due to fibroids, such as miscarriage, preterm labor, placenta previa, placental abruption, fetal growth restriction, malpresentation, and peripartum hemorrhage, may occur in 10 to 30% of these patients. 3 Moreover, the most common complications of fibroids during pregnancy are pain due to degeneration or torsion of the pedunculated fibroid, as well as pelvic pressure-related problems and vaginal bleeding. 4 5 The frequency of major adverse outcomes correlates with the size of the fibroid and is especially high in women with fibroids > 5 cm in diameter. 6 7

The majority of prospective studies using ultrasound to follow the size of uterine fibroids during pregnancy have shown that fibroid size remains stable (< 10% change in size) from the pregestational period to the end of pregnancy. 6 In spite of this, some studies report an increase in size during pregnancy. 8 Besides, larger fibroids (> 5 cm in diameter) are more likely to grow, whereas smaller fibroids are more likely to remain stable in size. 4 Fibroids may cause pregnancy loss but there is a lack of consensus in the association between uterine fibroids and recurrent miscarriages among the medical community. 9 Depending on the size and location, fibroids may alter the contour of the intrauterine cavity, leading to decidual atrophy or distortion of the vascular architecture of the decidua and affect implantation, placentation, and ongoing pregnancy. 10

Myomectomy during the course of cesarean section (CS) is questionable because of an increased risk of intrapartum and short-term postpartum complications, especially bleeding. However, many authors agree that myomectomy is a safe procedure during CS. 11 The recent literature advocates elective or opportunistic myomectomy in well-selected cases during CS. 12

Although there were various studies about pregnancies with fibroids in the literature, the number of studies on the effect of small fibroids on pregnancy was limited. Therefore, in the present study, we aimed to evaluate the obstetric outcomes of singleton high-risk pregnancies with a uterine fibroid < 5 cm.

## Methods

The present retrospective cohort study was conducted among high-risk pregnant women who were followed-up by a single surgeon (M. S. B.) at the Division of Perinatology, Department of Obstetrics and Gynecology from the Hacettepe University Hospital between August 2016 and December 2019. Women with uterine anomaly, those with multiple pregnancies, and pregnant women with fibroids > 5 cm in diameter or multiple fibroids were excluded. In the remaining 172 patients, pregnant women with preconceptionally diagnosed small/medium size (< 5 cm) single uterine fibroids (study group) were compared with pregnant women without uterine fibroids (control group). The required data were obtained from the patients' files and the electronic database of our institution. The study protocol was approved by the Ethics Committee of Hacettepe University with the reference number of GO 19/1064, and informed consent was obtained from all participants.

Pregnancies with poor obstetric history, chronic inflammatory diseases, autoimmune disorders, metabolic and/or inflammatory risk factors for placenta-mediated pregnancy complications were defined as high-risk pregnancy in this study. All high-risk pregnancies were included in a special antenatal care program for the optimal management of their risk factors. Pregnancy follow-up consisted of serial ultrasonography to evaluate fetal growth, aneuploidy screening (combined or triple test), fetal anatomy scanning at the 20 ^th^ to 24 ^th^ gestational weeks, oral glucose challenge test, and a non-stress test performed according to national and international guidelines. The iron supplement (30 mg) was given to all pregnant women daily.

The study and control groups were compared in terms of maternal age, gravidity, parity, Beksac Obstetric Index pregnancy (BOIp), miscarriage rate, hemoglobin (Hb) level at the first trimester, gestational age at birth, birth weight, 5 ^th^ minute Apgar score, fetal presentation, postdelivery Hb (8 hours after delivery), and delta Hb levels (the difference between the postdelivery and first trimester Hb levels). The BOI is a special obstetric index for the assessment of risk levels in pregnancies depending on their previous obstetric histories [(number of alive children + ( *n* /10))/Gravida]. The BOI value calculated in the preexisting pregnancy was defined as BOIp. 13 Beksac Obstetric Index is used widely in the literature for the comparison of risk levels for different patient groups. This index is used in many studies regarding various types of maternal risk factors. 14 15 Furthermore, characteristics of the uterine fibroid (size, type, and location), the growth rate of the fibroid during pregnancy, location of the placenta, obstetric complications due to fibroids, cesarean myomectomy rate, and delta fibroid size (the difference between the size of the fibroid during delivery and at the preconceptional period) were evaluated in the study group.

Statistical analyses were performed using the IBM SPSS statistics software, version 22.0 (IBM Corp., Armonk, NY, USA). Variables were investigated using visual (histograms, probability plots) and analytical methods (Shapiro-Wilk test) to determine the normality of distribution. As the data were not normally distributed, the Mann-Whitney U-test was performed for the comparison of continuous variables, and the chi-square test was performed for comparing categoric variables between the groups. A two-tailed *p* -value < 0.05 was considered statistically significant.

## Results

The present study included 172 high-risk pregnant women. There were 25 cases in the study group and 147 cases in the control group. Demographic features and clinical characteristics of both groups were summarized in Table 1 .

**Table 1:** Comparison of demographic features and clinical characteristics between groups

	Study group ( *n* = 25) (median, min–max)	Control group ( *n* = 147) (median, min–max)	*p* -value
Maternal age	35.00 (18–41)	33.00 (20–42)	0.055
Gravidity	3.00 (1–8)	3.00 (1–9)	0.522
Parity	2.00 (1–5)	2.00 (1–5)	0.819
BOIp	0.657 (0.22–1.31)	0.657 (0.15–1.31)	0.858
Miscarriage rate (n,%)	4 (16%)	25 (17%)	0.840
Hb level at 1 ^st^ trimester	12.3 (7.6–14.8)	12.4 (8.4–14.8)	0.531
Gestational age at birth	37.00 (34–38)	37.00 (34–40)	0.782
Birth weight	2,930 (2,300–3,720)	2,970 (810–4,310)	0.602
5 ^th^ minute Apgar score	9.00 (4–10)	10.00 (0–10)	0.693
Postdelivery Hb level	10.1 (5.6–12.2)	10.4 (6.3–13.6)	0.142
Delta-Hb level	2.0 (0.3–5.0)	1.8 (0.4–4.7)	0.263

Abbreviations: BOIp, Beksac Obstetric Index pregnancy; Hb, hemoglobin, min, minimum; max, maximum; n, number.

Miscarriage rates were similar between the groups (16% and 17% for the study and control groups, respectively [ *p* = 0.84]). There were no statistically significant differences between the groups in terms of gestational age at birth, birth weight, and 5 ^th^ minute Apgar score. There were no early preterm deliveries in the cohort. Five (20%) and 32 (21%) late preterm deliveries were detected in the study and control groups, respectively. Median BOIp was 0.657 for both groups ( *p* = 0.858). Malpresentation rates were 4% and 4.1% for the study and control groups, respectively ( *p* = 0.841). The number of patients who received a blood transfusion for postpartum anemia was 1 (4%) in the study group and 2 (1.4%) in the control group ( *p* = 0.351).

Basic characteristics of the study group were presented in Table 2 . None of the uterine fibroids was located in the cervix. Topographic locations of uterine fibroids were subserosal (68%), intramural (28%) and submucosal (4%). The mean size of uterine fibroids at preconception and birth was 2.16 cm (±0.75) and 2.54 cm (±0.77), respectively. Fifteen uterine fibroids (60% of the study group) were increased, and the others (40%) were stable in size during pregnancy. The mean delta fibroid size was 0.37 cm (±0.61).

**Table 2:** Basic characteristics of the study group (25 pregnant women with uterine fibroids)

Parameter	N (%) or mean ± SD
Uterine fibroid location	
Anterior	15 (60)
Posterior	7 (28)
Fundal	3 (12)
Uterine fibroid topographic location	
Subserosal	17 (68)
Intramural	7 (28)
Submucosal	1 (4)
Uterin fibroid size preconception (cm)	2.16 ± 0.75
Uterin fibroid size at birth (cm)	2.54 ± 0.77
Modes of delivery	
Miscarriage	4
Vaginal delivery	2 (9.5 ^‡^ )
CS without myomectomy	4 (19 ^‡^ )
CS myomectomy	15 (71.4 ^‡^ )

Abbreviations: CS, cesarean section; N, number; SD, standard deviation.

‡ Rates are given after exclusion of cases with miscarriage.

Placental locations were anterior (32%), posterior (48%), and fundal (%20) in the study group. Placenta previa and placental abruption were not shown in the study group. Five patients from the study group (20%) had retroplacental myoma. Out of these five patients, only one was complicated with preterm delivery, and the remainders had no adverse pregnancy outcome. Miscarriage was shown in four cases in the study group. Two patients were hospitalized with pelvic pain due to degeneration of the fibroid, and three patients were hospitalized with vaginal bleeding in the first trimester. The frequency of preterm delivery was 20% among women with fibroids. In the study group, the CS rate was 90.5%, and none of the CS was performed due to uterine fibroids. Myomectomy was performed during CS in 15 cases (71.4%).

## Discussion

Uterine fibroids are the most common benign uterine tumors, with an estimated incidence of 20 to 40% in women during their reproductive years. The association of myoma and pregnancy is becoming more frequent due to the advanced maternal age. 16 In our study, 14.5% of 172 pregnant women had uterine fibroids with a diameter < 5 cm.

Previous studies have shown a possible association between fibroids and increased risk of adverse pregnancy outcomes. 17 18 19 In 2008, Klatsky et al 18 reported an increased risk of miscarriage in women with uterine fibroids compared with women without fibroids. According to a study conducted by Navid et al, 20 the frequency of miscarriage among women with fibroids was 10%. In our study, we found that miscarriage rates were 16% and 17% in women with and without myomas, respectively, most probably due to the characteristics of our cases. Fetal malpresentation has also been reported to be more common among women with fibroids. Klatsky et al 18 reported a frequency of malpresentation of 16% among women with fibroids, ∼ 2.5 times higher than in the general population. Similar results have been reported by Navid et al 20 In our study, malpresentation rates were 4% and 4.1% for the study and control groups, respectively. This may be explained by the inclusion of the patients with a smaller size of fibroids. This shows us that small fibroids do not affect the malpresentation rate. Shavell et al 21 showed that compared with women with no fibroids or small fibroids (≤ 5 cm), women with large fibroids (> 5 cm) delivered at a significantly earlier gestational age (38.6 versus 38.4 versus 36.5 weeks). According to our results, the preterm delivery rate was 20% in the study group, and there was no statistically significant difference between the groups in terms of gestational age at birth (median gestational age was 37.0 weeks for both groups). Placental abruption has been associated with uterine fibroids and seems to be related to fibroid location. 22 Placental abruption and placenta previa were not observed in our study group, most probably due to the size of the fibroids. Fetal growth does not appear to be affected by the presence of uterine fibroids, which we have also demonstrated in our study. 22 Degeneration occurs in around 10% of pregnant women with fibroids. 4 Likewise, two patients (8%) were hospitalized with pelvic pain due to degeneration in our study and none of these pregnancies complicated with any adverse event.

Risk factors for pregnancy complications appear to be the size and the location of fibroids, such as the large size of over 5 cm and retroplacental location and/or distortion of the uterine cavity. 17 18 19 20 21 22 23 In our study, we have demonstrated that fibroids < 5 cm did not provide an additional risk in terms of adverse pregnancy outcomes. The risk of uterine fibroid-related complications during pregnancy might be primarily correlated with the size of myomas.

Pregnancy-related increases in steroid hormone levels and uterine blood flow affect fibroid growth. 4 Aharoni et al 24 reported leiomyomas to be mostly unchanged during pregnancy (59%). It has also been reported that the size of the fibroids was increased in 22% of the patients and the growth percentage of these fibroids was found to be 25%. 24 In our study, the size of the fibroids was increased in 60% of the cases, and the growth percentage of the fibroids was also 25%.

Song et al 25 reviewed 9 case-control studies that included more than 1,000 women with ﬁbroids, of whom 41% underwent cesarean myomectomy and 59% underwent CS alone. They could not demonstrate any difference between groups in terms of safety parameters. 25 Turgal et al 11 found no statistical difference in the adhesion formations between women who had previously undergone cesarean myomectomy for small ﬁbroids and controls who had not undergone myomectomy during their previous CS. Cesarean sections of our patients were performed due to other obstetrical indications, and we have demonstrated that opportunistic cesarean myomectomy was convenient as a safe and viable option in well-selected cases. Thus, we may conclude that cesarean myomectomy may be safely performed in patients with a myoma < 5 cm by experienced physicians. However, appropriate case selection must still be performed and previously reported complications must be kept in mind.

The limitations of this study were the relatively small number of cases, retrospective design, and lack of information related to fibroid sizes throughout gestational trimesters. On the other hand, longitudinal follow-up of the cases and presentation of single surgeon experience are the strengths of this study.

## Conclusion

In conclusion, small size uterine fibroids (< 5 cm) seem to have no adverse effect on pregnancy outcomes even in high-risk pregnancies, and cesarean myomectomy may be safely performed in properly selected cases.
